# The role of healthy diet in the prevention of osteoporosis in perimenopausal period

**DOI:** 10.12669/pjms.304.4577

**Published:** 2014

**Authors:** Małgorzata Kostecka

**Affiliations:** 1 Małgorzata Kostecka, Department of Food and Biotechnology, Faculty of Food Science and Biotechnology, University of Life Science, Lublin, Poland.

**Keywords:** Bone formation, Calcium, Diet, Osteoporosis, Phosphorus, Vitamin D

## Abstract

***Objective:*** The aim of the study was to assess the diet for its effect on normal bone mineralization and qualitative analysis of dietary supplements used in the prevention of osteoporosis.

***Methods:*** Research data were acquired based on a questionnaire developed by the author. A 24-hour diet recall method was used with the involvement of Dieta 5.0 software developed by the National Food and Nutrition Institute in Warsaw in Poland. The second part of the study involved an analysis of the most popular dietary supplements available over the counter. Complexometric titration was used to separate and identify calcium.

***Results:*** The results of a 24-hour diet recall indicate that 47% of the respondents consumed 550-750 mg of calcium daily, and only 21% of the subjects consumed more than 1200 mg of calcium every day. The results give cause for concern: none of the analyzed diets supplied the recommended daily amounts of vitamin D, and only 29% of the respondents admitted taking vitamin D supplements. The results of the survey indicate that consumers have insufficient knowledge about lifestyle diseases, including osteoporosis.

***Conclusion:*** Diet of large part of society is not properly balanced which can cause abnormalities in achieving proper bone mineralization. Long-term deficiencies in calcium and vitamin D in daily diet are the cause for taking dietary supplements. Unfortunately, some preparations on the market do not have adequate storage. It happens that these preparations are poorly absorbed and the amount of active compound is too low. Changes in the nutritional regimen are required already during childhood because nutritional mistakes are the main cause of diet-related diseases in adulthood.

## INTRODUCTION

Osteoporosis is a skeletal disease characterized by low bone mass and disrupted bone architecture which increases bone brittleness and the risk of fracture.^1^ The loss of osseous tissue and bone lamellae in spongy bone proceeds faster than bone formation, leading to the loss of bone flexibility and stability. The loss of bone mass often proceeds unnoticed, it is a continuous process which may not produce any symptoms. Bone mineralization is significantly affected by calcium and phosphorus metabolism which is controlled by vitamin D, parathormone and calcitonin.^2^ Osteoblast and osteoclast activity is regulated by cytokinins, estrogen and testosterone. After the age of 40, physiological loss of bone mass is observed, and the relationship between organic collagenous fibers and inorganic salts becomes altered.^3^

Until recently, it was believed that osteoporosis affects mainly post-menopausal women and elderly men. The results of recent research however, indicate that osteoporosis may occur in people older than 40 as well as in youths. The disease is more likely to affect women than men, and it is more prevalent in Caucasian and Asian females. Other contributing factors include low estrogen levels, genetic predispositions, low body weight, smoking, excessive consumption of coffee and long periods of immobilization.^4^ Dietary factors which increase the risk of osteoporosis include a deficiency of calcium, 1,25-dihydroxyvitamin D and protein.^1^ Osteoporosis prevention should begin early in childhood through the consumption of calcium-rich foods such as milk and dairy products to promote optimal bone formation.^5^

The calcium to phosphorus ratio is also an important consideration, but the modern diet rarely guarantees the right balance between those nutrients. The presence of phosphates in food products (stabilizers, anticaking agents, emulsifiers and pH stabilizers)^6^^,^^7^ increases phosphorus concentrations in the diet which leads to the acidification of bodily tissues and intensified parathyroid activity. Parathyroid glands produce parathormone which promotes calcium loss from teeth and bones. Vitamin D is essential for maintaining the calcium-phosphorus balance in the body and mineralizing the bone matrix. A vitamin D deficiency may have a detrimental effect on bone quality and calcium absorption from food.^8^ European consumers have a generally low level of awareness about osteoporosis. The majority of the population does not recognize the link between osteoporosis, lifestyle and nutrition.

In the last decade, the consumption of milk and dairy products has decreased by nearly 20%, whereas the proportion of highly processed foods and confectionary products in Polish consumers' diets increased by more than 25% (estimate of the Department of Nutritional Economics of the National Food and Nutrition Institute in Warsaw based on an unpublished survey of household budgets carried out by the Central Statistical Office in 2008). The growing popularity of dietary supplements, including calcium, leads to unlimited consumption of minerals and vitamins, often without medical consultation. In this study we aimed to assess the diet for its effect on normal bone mineralization and qualitative analysis of dietary supplements used in the prevention of osteoporosis

## METHODS

Research data was acquired based on a questionnaire developed by the author. The questionnaire was distributed among patients of the First Clinical Hospital in Lublin, in hospital in Rzeszów and in clinic in Kraśnik in Poland between November 2011 and January 2012. In the study take part 430 respondents. All participants were women aged 45 to 65 years. A 24-hour diet recall method was used with the involvement of Dieta 5.0 software developed by the National Food and Nutrition Institute in Warsaw.

The second part of the study involved an analysis of the dietary supplements available over the counter in most European countries. Complexometric titration was used to separate and identify calcium. Supplement tablets were weighed to the nearest 0.0001g and ground in a ceramic mortar. Analytical samples of approximately 0.5000g were prepared. The substance was dissolved in a flask containing 100 ml distilled water. A 10ml sample of the solution was transferred to an Erlenmeyer flask, and Eriochrome T black was added as an indicator. The sample was analyzed by titration with a sodium versenate solution. All measurements were performed in three replications. The analyses were carried out in an aqueous environment, with the addition of artificial gastric juice (pH=2) and artificial intestinal juice (pH=8). The results were used to determine the calcium content of the analyzed supplements per tablet.

## RESULTS

An analysis of survey results revealed that only 1/3 of the respondents evaluated their health condition as satisfactory, and they were not affected by chronic diseases. Excessive body weight was the second most prevalent issue diagnosed in the survey. In the studied population, 25% of the respondents were overweight and 13% were obese. Osteoporosis had been diagnosed in 21% of the polled subjects (women aged 55-60 years accounted for 75% of all diagnosed with osteoporosis). (Table I)

The surveyed respondents were of the opinion that they had a very high level of nutritional knowledge. 56% of the polled subjects were highly familiar with calcium-rich foods, whereas 37% had a fair knowledge of the above. Only 7% of the respondents were unfamiliar with the composition of popular food products. (Table-II). The majority of subjects did not use their diets in accordance with healthy nutrition principles. 

The results of a 24-hour diet recall indicated that 47% of the respondents consumed 550-750 mg of calcium daily, and only 21% of the subjects consumed more than 1200 mg of calcium every day. Average calcium content of the diet was 871mg (SD 131 mg). This problem requires a detailed analysis, because a postmenopausal women who don’t using estrogen therapy, and all women after 65 years old should consume 1500 mg calcium/day.^9^ Vitamin D levels were also determined in the analyzed diets. The results give cause for concern: none of the analyzed diets supplied the recommended daily amounts of vitamin D, and only 29% of the respondents admitted taking vitamin D supplements. 

The contemporary diet is also rich in phosphorus which limits calcium absorption. The diets of 74% of the respondents supplied 98-176% of the recommended daily amounts of phosphorus. Only14% of the surveyed cases, diet met the demand for phosphorus according to age and sex. General practitioners play a very important role in educating consumers about healthy nutrition. People with a high risk of osteoporosis should be referred to dieticians, and general practitioners should advise their patients on the type, amount and safety of dietary supplements.

Dozens of calcium-containing supplements are easily available over the counter and can be consumed without medical consultation. The results of the survey indicate that 47% of the respondents buy calcium supplements, and nearly 75% of them use them regularly and at least once daily. Supplements containing calcium in their composition are widely advertised as an ideal way to supplement calcium deficiency, and the recommended dose ranges from1000 to 1500mg per day. The quantitative composition of the majority of the examined dietary supplements was consistent with the information declared by the manufacturer on the packaging. The determined content of ions (mg) did not differ from that given by the supplier by more than 5-10% (Table-III). Significant differences were noted in two products: supplement 3, and supplement 6. In samples containing artificial gastric juice, which imitates the acidic environment of preliminary digestion in the stomach, the active substance content increased by 10%, indicating that calcium carbonate is more readily absorbed at this stage of digestion. In samples containing artificial intestinal juice, the availability of calcium ions in a base environment (57.2%) was marked by a further decrease. According to the supplement 6 (prolonged-release enterosoluble capsules) contains the highest calcium concentrations on the market. In this case, the active ingredient is also poorly absorbed calcium carbonate, and capsule coating contains shellac which prevents the capsule from being effectively digested by gastric or intestinal juice. Products containing Ca^2+^ ions in the form of organic salts, including citrate and gluconate, and chelated calcium ions are characterized by the highest bioavailability.^7^^,^^10^

## DISCUSSION

The study showed that the typical diet does not cover the demand for nutrients, vitamins and minerals. Especially important is the deficiency of calcium and vitamin D, because it can promote reduction in bone mineral density. The deficit of calcium in the diet is due to improper diet and ignorance of consumers. The respondents' declared level of knowledge was not confirmed by their answers regarding food products chosen to enrich their diets with calcium. A typical European diet is rich in animal protein, and high consumption of salt, alcohol and caffeine reduces calcium absorption and increases calcium excretion. In Asian countries the diet is enriched vegetable protein, although it is too rich in calcium and vitamin D.

**Table-I:** The age structure of women participating in the study and the incidence of osteoporosis

*Age*	*45-50*	*50-55*	*55-60*	*60-65*
**Number of women in study**	**130**	**90**	**150**	**60**
**Number of women with osteoporosis in study**	**-**	**4**	**68**	**18**

**Table-II:** What products do you consume at least once every week to enrich your diet with calcium?

***Product***	***Respondents %***	***Product***	***Respondents %***
Milk	34%	Blue cheese	28%
Curd cheese	47%	Cream	45%
Yoghurt	39%	Soy	12%
Acidophilus milk	4%	Sprats	17%
Kefir	17%	Sardines	24%
Buttermilk	23%	Nuts	19%
Cream cheese	61%	Cruciferous vegetables	39%

**Table-III:** Calcium ion content of dietary supplements

	***Dietary supplement***	***Mass of Ca*** ^2+^ ***ions in three replicates (mg)***			***Average content of Ca*** ^2+ ^ ***(mg)***	***Ca*** ^2+ ^ ***content declared by the manufacturer (mg)***
		***1***	***2***	***3***		
	Aqueous environment					
1	Supplement 1	255	257	261	257.6	250 mg
2	Supplement 2	467	478	470	471.6	500 mg
3	Supplement 3	343	354	348	348.3	500 mg
4	Supplement 4	245,5	247	246	246.1	250 mg
5	Supplement 5	502	497	496	498.3	500 mg
6	Supplement 6	376	379	377	377.3	600 mg
7	Supplement 7	178	177	175.5	176.8	180 mg
	Artificial gastric juice, pH=2					
1	Supplement 1	243	241	244	242.6	250 mg
2	Supplement 2	454	455	459	456	500 mg
3	Supplement 3	395	397	395	395.6	500 mg
4	Supplement 4	249	246	248	247.6	250 mg
5	Supplement 5	499	489	492	493.3	500 mg
6	Supplement 6	256	254	251	253.6	600 mg
7	Supplement 7	179	176	178	177.6	180 mg
	Artificial intestinal juice, pH=8					
1	Supplement 3	289	284	286	286.3	500 mg
2	Supplement 6	288	291	289	289.3	600 mg

The vast majority of the respondents (83%) had practically no knowledge about the role of vitamin D in maintaining a healthy calcium balance, and 15% of the polled subjects believed that vitamin D is vital only during infancy. The vitamin D receptor (VDR) gene is thought to be a candidate gene for osteoporosis.^11^ Research shows that proper supply of multi-role of vitamin D in combination with a diet rich in calcium can be crucial in the prevention of osteoporosis. Chinese-American women have a high risk of osteoporosis. Increasing consumption of calcium-rich foods, many of which are also fortified with vitamin D, is a safe way to increase their calcium and vitamin D intake.^12^ People with a high risk of osteoporosis should be referred to dieticians, and general practitioners should advise their patients on the type, amount and safety of dietary supplements.

The problem of meeting the demand for calcium is present in patients suffering from gastrointestinal diseases, particularly of the intestines. Impaired absorption and drugs significantly reduce the absorption of calcium, which is a risk factor for osteoporosis. Self-reported lactose intolerance, leading to dietary restrictions, is the single major determinant of low calcium intake. Inadequate calcium intake is present in one third of inflammatory bowel disease patients.^13^

Osteoporosis is a chronic disease affecting millions of people worldwide. It is generally accepted that acquisition of a high peak bone mass (PBM) early in life can reduce the risk of osteoporosis later in life. But the problem of ensuring the correct calcium intake derived from a balanced diet is very difficult to solve. Contemporary lifestyle further reduces its bioavailability from the diet. Natural calcium is well absorbed and cause irregular heart of gold and does not increase the likelihood of developing coronary heart disease. Changes in the nutritional regimen are required already during childhood because nutritional mistakes are the main cause of diet-related diseases in adulthood, such as obesity, osteoporosis, cancer and cardiovascular diseases.^14^^,^^15^ A balanced diet containing a healthy ratio of natural calcium to phosphorus and the recommended levels of vitamin D contributes to bone mineralization and high bone density, including during the menopause. The Polish study on diet of children aged 10-12 years showed that in 46% of the respondents Insufficient intake of calcium, while fully covering the demand for phosphorus, resulted in improper ratio of these minerals in the diet (0.62 against the recommended. 1). This result suggests that in the adulthood, these children will have a greater risk of developing osteoporosis.^16^

Nonpharmacologic strategies should always be implemented, but many patients also need pharmacologic intervention to achieve adequate fracture protection. It is evident today that although low bone mineral density (BMD) is an important determinant of bone fragility, it is not the only one, hence, drugs used in the treatment of osteoporosis must not only show to promote changes in BMD, but reduce the incidence of fractures. Safety issues should be always considered on an individual basis.^17^ Calcium is an essential nutrient for skeletal health however it has been suggested that supplemental calcium may be associated with adverse cardiovascular effects, raising widespread concern about their use. One suggested mechanism is via increasing carotid atherosclerosis.^18^ Numerous clinical studies have been performed to determine the effect of calcium on arterial calcification. Therefore, the use of preparations containing high doses of calcium in the elderly should be undertaken under medical supervision. These people are usually likely to develop coronary artery disease and atherosclerosis are developed.

When using supplements it is important to know which organic or inorganic calcium occurs. As for the absorption studies show that performance is similar for all salts of calcium, regardless of the water solubility. However, it is slightly smaller than the absorption capacity of calcium intake from diet. Supplementation cannot replace proper nutrition. Natural calcium derived from food has better effect on bone mineral density than supplements.^19^

Czernichow in the study of the French population has obtained very similar results for the average calcium intake from diet to the results of the Polish population. The average daily dietary calcium intake was 966.4 mg. Calcium supplements were taken by 38% of participants and older patients tended to take more. Limitations of their study included convenience sampling and patient self-report. Daily vitamin D intake among this sample of postmenopausal osteoporotic women in France was significantly lower than recommended dosages. At least 50% of these patients might benefit by adding vitamin D to their current therapy.^20^

Recent studies also show the problem of the Spanish supplementation with calcium and vitamin D in patients at risk for developing osteoporosis. Of those participants who derived < 800 mg/day of calcium from their diet, 67% reported taking a multivitamin with calcium and 50% reported taking supplemental calcium alone. Similar incidences of multivitamin with calcium, and supplemental calcium use alone were seen in participants obtaining 800–1200 mg/day and ≥1200 mg/day from their diet.^21^ The use of supplementation with a well-balanced diet leads to excessive calcium intake.

Future studies are needed to better define the optimal use of vitamin D supplements in promoting bone health and preventing osteoporotic fractures. Some previous studies have failed to demonstrate a significant benefit for vitamin D supplementation on fracture risk.^22^^,^^23^ For example, in a study of postmenopausal women recruited to a Women’s Health Initiative trial with or without osteoporosis, calcium plus vitamin D supplementation increased hip bone mineral density.^21^ However, a significant effect of supplementation on the incidence of hip fracture was observed only after data from non-treatment-compliant subjects was analyzed.

The New U.S. Preventive Services Task Force (USPSTF) recommends against daily supplementation with 400 IU or less of vitamin D3 and 1000 mg or less of calcium for the primary prevention of fractures in noninstitutionalized postmenopausal women. This recommendation does not apply to the treatment of persons with osteoporosis or vitamin D deficiency.^24^

Dietary supplement marketed in Poland and the European Union are not subject to stringent qualitative or quantitative control. Many products do not contain minerals and vitamins of declared purity or in the claimed amount. Consumers who use such products are not only unable to derive health benefits, but they are also at the risk of experiencing dangerous interactions. In addition, supplementation should be used in cases where the diet cannot cover the demand for minerals and vitamins, and after recommendation from a doctor.

## Notes

***Funding: ***The work was financed by public funds for science and higher education.
